# Editorial: Vaccines and therapeutics utilizing new adjuvants and potential inhibitors to target emerging infectious diseases

**DOI:** 10.3389/fimmu.2025.1716739

**Published:** 2025-11-11

**Authors:** Jiae Kim, Ousman Jobe, Mara Jana Broadhurst

**Affiliations:** 1 United States Military HIV Research Program, Henry M. Jackson Foundation for the Advancement of Military Medicine, Bethesda, MD, United States; 2 Laboratory of Adjuvant and Antigen Research, United States Military HIV Research Program, Center for Infectious Disease Research, Walter Reed Army Institute of Research, Silver Spring, MD, United States; 3 Department of Pathology, Microbiology, and Immunology, College of Medicine, University of Nebraska Medical Center, Omaha, NE, United States

**Keywords:** emerging infectious diseases (EIDs), food and water borne diseases, viral infections, in silico, adjuvants, vaccine delivery platforms, computational approach and techniques, bacterial infections

Infectious diseases that are persistent or increasing in incidence continue to have a broad impact on populations worldwide. Emerging infectious diseases often pose an outsized burden on health systems due to limited medical countermeasures and minimal population immunity. The catastrophic health burdens of emerging infectious diseases highlight the need for innovative strategies to develop effective preventive and therapeutic interventions.

This Research Topic “*Vaccines and therapeutics utilizing new adjuvants and potential inhibitors to target emerging infectious diseases*” comprises 10 articles that address critical emerging pathogens including SARS-CoV-2 (Sui et al., Xu et al., Neville et al.), influenza A virus (Zhang et al.), *Neisseria gonorrhoeae* (Lu et al.), *Salmonella enterica serovar Rissen* (Cuomo et al.), novel adjuvant effect on immune responses (Shamseldin et al.), and *in silico* and computational approaches for vaccine development and targeting (Panda et al.).

We have seen the impact of innovative vaccine platforms against emerging viruses with the implementation and utilization of SARS-CoV-2 vaccines. Xu et al. performed a prospective, longitudinal study of a healthcare worker immunization program in China, demonstrating short-lived neutralizing antibodies following COVID-19 booster immunization with incomplete protection from breakthrough infection. Therefore, although these vaccines are indeed effective, further improvements in the boosting strategy are warranted. Sui et al. examined whether boosting intranasally (i.n.) with an adjuvanted subunit vaccine would protect against a SARS-CoV-2 challenge. Their work demonstrated the efficacy of boosting with an i.n. formulation, showing that lower oral and lung viral loads were correlated with mucosal ACE2 inhibition activity. Neville et al. examined the utilization of a peptidomimetic of complement C5a, EP67, with inactivated SARS-CoV-2, which resulted in enhanced immune responses. This work also demonstrated the possible utility of EP67 on its own as a potential antiviral agent.

Emerging avian influenza viruses pose persistent public health threats. Zhang et al. developed a vaccine against Eurasian avian-like H1N1 (EA H1N1) influenza. This subunit influenza vaccine formulation contained adjuvant gram-positive enhancer matrix (GEM) particles derived from *Lactococcus lactis*. Intranasal administration or co-administration via the i.n. and intramuscular (i.m.) routes generated mucosal and Th1-biased immune responses that were inferior to the responses induced by i.m. alone and displayed undetectable viral titers in the lungs after challenge. This study provides information on possible platforms and routes of administration for vaccination that are not only applicable to the viruses examined but perhaps other viral infections.

Bacterial infections are the cause of a wide range of pathogenic infections, from sexually transmitted diseases to foodborne illnesses. The emergence of multidrug resistance makes these emerging pathogens of importance in public health. Lu et al. examined the immunogenicity and efficacy of a trivalent vaccine targeting *Neisseria gonorrhoeae*. This formulation induced stronger circulating IgG and IgA antibody responses in mice compared to monovalent vaccine formulations. The serum from the vaccinees killed various strains of *N. gonorrhoeae in vitro*; however, it was only moderately effective in a mouse intravaginal challenge model. These results indicate the potential utility of a multivalent vaccine formulation strategy, especially against multidrug-resistant strains. Cuomo et al. examined modifications to the lipid A of *Salmonella enterica* serovar Rissen (S. Rissen) that render the bacteria phage-resistant. The modifications to lipid A were utilized in a potential lipopolysaccharide (LPS)-based vaccine, which was demonstrated to be less toxic and could be effective against salmonellosis.

*In silico* and computational work are new approaches to generating and developing novel vaccine formulations. Panda et al. utilized these approaches to develop a potential vaccine targeting Scrub typhus, which is a life-threatening illness caused by the gram-negative bacterium *Orientia tsutsugamushi*. The research group used a reverse vaccinology approach by taking subunit candidates and determining a possible formulation for a vaccine. Through molecular docking and simulations, the final construct was found to show high antigenicity, stability, and solubility. Sethi et al. also utilized an immunoinformatics approach to develop a vaccine formulation against *Leptospira*. This formulation was designed to be a multi-epitope subunit vaccine (MESV) with an adjuvant, and when tested *in silico*, it was shown to elicit robust B and T cell responses. An immunoinformatics approach to designing a multi-epitope subunit vaccine may provide additional tools to enhance vaccine development. It will be interesting to see if these two promising candidates can be effective not only *in silico* but experimentally on the bench.

Adjuvants are a critical component of vaccines, and there is a requirement for the development and understanding of new adjuvants. Shamseldin et al. provided more mechanistic insight into a potential adjuvant, Bordetella colonization factor (BcfA), an outer membrane protein. This adjuvant has been demonstrated to activate Th1/Th17 immune responses. The work examined BcfA in the context of antigen-presenting cells, bone marrow-derived dendritic cells, and human PBMCs. Informing the vaccinology field on the mechanism of adjuvant action and how an adjuvant activates immune responses is critical in aiding the development of vaccine formulations that target emerging infectious diseases.

Finally, a review written by Thom and D’Elia introduced and described the idea of Host-Directed Therapies (HDTs) as an alternative approach to pathogen-targeting therapeutics against emerging infectious diseases. This work explores the notion of targeting pathways to diminish the host response to a pathogen.

New technologies and approaches are necessary for both preventative vaccines and therapeutics against emerging infectious diseases, which continue to impact populations on a global scale. By utilizing bench-side and *in silico* approaches, these various platforms could make it to the clinic in the future. The contributions to this Research Topic highlight several new possible platforms and opportunities for targeting these diseases. We thank the authors of the articles for their contributions.

